# IL-33/ST2 axis mediates diesel exhaust particles-induced mast cell activation

**DOI:** 10.1186/s10020-024-01035-y

**Published:** 2024-12-20

**Authors:** Wun-Hao Cheng, Ting-Li Zhuang, Meng-Jung Lee, Chun-Liang Chou, Bing-Chang Chen, Han-Pin Kuo, Chih-Ming Weng

**Affiliations:** 1 School of Respiratory Therapy, Taipei Medical University College of Medicine, 250 Wu-Hsing Street, Taipei, 11031 Taiwan; 2 Respiratory Therapy, Division of Pulmonary Medicine, Department of Internal Medicine, Wan Fang Hospital, Taipei Medical University, Taipei, Taiwan; 3 Pulmonary Medicine Research Center, Taipei Medical University, Taipei, Taiwan; 4 Department of Thoracic Medicine, Taipei Medical University Hospital, Taipei, Taiwan; 5 Research Center of Thoracic Medicine and Asthma, Taipei Medical University Hospital, Taipei, Taiwan

**Keywords:** Airborne pollutant, Aryl hydrocarbon receptor, IL-33, Mast cell

## Abstract

**Background:**

Mast cells are implicated in the pathogenesis and severity of asthma in children and adults. The release of proinflammatory mediators and cytokines from activated mast cells (MC) is associated with Type 2 (T2) cell-skewed inflammation.

**Methods:**

We obtained the airway tissues of Balb/c mice with or without intra-tracheal diesel exhaust particles (DEP) instillation to measure the extent of tryptase^+^ MCs infiltration and interleukin (IL)-33 expression. Cultured human mast cells (HMC-1) were stimulated with DEP to determine the role of aryl hydrocarbon receptor (AhR) in mediating the synthesis and release of IL-33 and type-2 cytokines.

**Results:**

In the control animals, most of the MC accumulated in the submucosal vessels without expression of IL-33. Intra-tracheal DEP installation increased the number of IL-33^+ ^MC infiltrating in the epithelial and sub-epithelial areas of mice. Human MC exposed to DEP upregulated mRNA and protein expression of IL-33. These effects were abolished by knockdown of expression of the AhR or AhR nuclear translocator (ARNT) by small interfering (si)RNA transfection. DEP also activated nuclear factor-kappa B (NF-κB) to facilitate nuclear translocation of the AhR. DEP increased MC migration and induced the synthesis and release of IL-4, IL-5, and IL-13 in MCs, and these effects were abolished by anti-ST2 antibodies.

**Conclusions:**

Airborne pollutants may activate MCs to produce IL-33 via the AhR/NF-κB pathway, leading to type 2 cytokines production and enhancing MC airway epithelium-shifted migration through the autocrine or paracrine IL-33/ST2 axis.

## Background

Asthma is a chronic airway inflammation disease. This process involves the activation and differentiation of Type 2 (T2)-related cells, which release IL-4, IL-5, and IL-13, thus exacerbating asthma (Gauvreau et al. 2023). Mast cells (MC) have been implicated in the pathogenesis and severity of asthma in children and adults (Diamant et al. 2007). MC can be found adjacent to blood vessels in the lamina propria of the human airway mucosa. Interestingly, in asthmatics, MC also migrates to other structures, such as the airway epithelium, mucous glands, and airway smooth muscle (Reuter et al. 2010). The increased MC number in asthma patients is associated with evidence of T2 cell-skewed inflammation (Bergqvist et al. 2015).

Studies have revealed that airway epithelium-derived cytokines, such as IL-33, serve a central role in T2 inflammation in asthma (Hallstrand et al. 2014; Iijima et al. 2014; Nagarkar et al. 2012). IL-33 induces eosinophilic inflammation and goblet cell hyperplasia in OVA-induced asthma mice model (Ishinaga et al. 2017; Stolarski et al. 2010), and IL-33/ST2 axis blockage reduced the total cell and eosinophil count in BALF of allergic asthma (Lee et al. 2014). Several studies have shown that abundantly expressed IL-33 in asthma patients (Prefontaine et al. 2010, Raeiszadeh et al. 2014) and OVA challenge experimental mice model (Louten et al. 2011) and confirmed elevated IL-33 expression in the airway epithelium of asthma patients (Prefontaine et al. 2010). IL-33 mediated signaling pathway further activates MCs, inducing MC migration, maturation, and cytokines production (Enoksson et al. 2011; Iikura et al. 2007; Saluja et al. 2015). This suggests that IL-33 is an important mediator implicated in mast cell-epithelium crosstalk in asthma.

Exposure to polycyclic aromatic hydrocarbons (PAHs) in airborne pollutants is a major healthcare concern. The aryl hydrocarbon receptor (AhR) has been identified as a mediator of the induction of toxicity elicited by halogenated aromatic hydrocarbons, including environmental toxins and PAHs (Gasiewicz et al. 2008). The AhR is a ligand-activated transcription factor and heterodimerizes with the aryl hydrocarbon receptor nuclear translocator (ARNT), then translocates from the cytosol to the nucleus and binds to dioxin response elements (DRE) (Mimura et al. 2003). The AhR has modulated the acute and late responses of MC in patients with chronic obstructive pulmonary disease (Sibilano et al. 2012). AhR activation induces the generation of reactive oxygen species (Kopf et al. 2010), which leads to MC-dependent inflammatory processes (Swindle et al. 2007). Our previous study demonstrated that activation of AhR leads to epithelium-derived alarmins release in airway epithelial cells of severe allergic asthma (Weng et al. 2018b). Air pollutants, such as DEP, also directly induce mast cell degranulation (Diaz-Sanchez et al. 2000). However, the role of the IL-33/ST2 axis in DEP-induced mast cell activation remains unclear.

Here, we showed that DEP exposure increased MC accumulation in the epithelium and lamina propria of the airway mucosa in experimental animals. Human MCs exposed to DEP synthesized and released IL-33 through an AhR activation pathway. IL-33/ST2 axis mediated MC migration and production of T2 cytokines in both paracrine or autocrine manners. Additionally, the molecular mechanisms underlying DEP-induced IL-33 production by MCs were explored.

## Methods

### Ethical approval of the study protocol

The protocol involving experimental animals was approved (Institutional Animal Care and Use Committee number: LAC-2020-0397) by the Animal Care and Use Committee of Taipei Medical University (Taipei, Taiwan).

### DEP preparation

The DEP (Standard Reference Material 2975) used in the present study was obtained from the National Institute of Standards and Technology (NIST, Gaithersburg, MD, USA), which is commonly used for air pollutant-related studies. The DEP was suspended in phosphate-buffered saline with 0.05% tween 80 and sonicated by ultrasonic disrupter before being used in an animal model or mast cell line. Well-suspended DEP consisting of the majority of particles being mean diameter of 20 nm. DEP contained 73.3% carbon (C), 15.9% nitrogen (N), 8.5% oxygen (O), 0.3% sulfur (S), 1.2% copper (Cu), and 0.8% zinc (Zn), and was spherical and aggregated (Bai et al. 2018).

### Mouse model of DEP challenge

Balb/c mice received intratracheal instillation of DEP or vehicle and were sacrificed 24 h later. The tryptase level was measured by immunohistochemical (IHC) staining, and the level of IL-33 expression was measured by immunofluorescence staining.

### Immunohistochemical and immunofluorescence staining

Sections of paraffin-embedded airway tissues of mice were immunostained with specific antibodies for tryptase or IL-33. In brief, lung tissue sections from mice were stained using specific antibodies against tryptase (Abcam, Cambridge, UK) or non-immune IgG (Santa Cruz, CA, USA) antibodies, respectively, followed by the use of an IHC kit (HRP/DAB Secondary Detection System; Millipore, Bedford, MA, USA). The fluorescence staining was performed using double staining for tryptase and IL-33, and Hoechst33342 was used as a counterstain. Images were acquired with an optical microscope (Olympus, Tokyo, Japan) or confocal microscope (Leica, IL, USA). The images were analyzed by ImageJ software (Bio-Rad Laboratories, Hercules, CA, USA).

### Cell lines

A human MC line (HMC-1) was kindly provided by Professor Shau-Ku Huang (Distinguished Investigator, National Institute of Environmental Health Sciences, National Health Research Institutes, Taiwan). Cells were maintained in Iscove's modified Dulbecco’s medium (Gibco, Grand Island, NY, USA) with 10% heat-inactivated fetal bovine serum, glutamine (2 mM), penicillin (100 U/mL), and streptomycin (0.1 mg/mL), all from Invitrogen (Carlsbad, CA, USA), in an incubator in an atmosphere of 5% CO_2_ at 37 °C. Typically, cells were seeded and maintained in culture plates of diameter 6 cm or 10 cm (Costar, Corning, NY, USA) for 24 h before stimulation with DEP (0–10 μg/mL). Before exposure to different concentrations of DEP, cultured cells were pretreated with small interfering (si)RNA or with specific inhibitors, such as CH223191 (a ligand-selective antagonist of the AhR, selleckchem, TX, USA) or PDTC (Sigma-Aldrich, MO, USA), to examine if responses were mediated through the AhR.

### siRNA transfection

Human mast cells (HMC-1, 1 × 10^5^ cells per well) were transfected with siRNA against AhR (si*AhR*) or scramble siRNA (negative control for AhR siRNA) (Dharmacon, UK) using DharmaFect 1 transfection reagent for overnight, and followed by stimulated with DEP for another 6 h. Then, cells were harvested for further study.

### Assay to measure MC migration

MC migration was analyzed in vitro (Sivalenka et al. 2004). In brief, HMC-1 cells (5 × 10^5^) were seeded in the upper chamber of a Transwell™ plate (pore size = 8 μm; Corning, NY, USA) with culture medium (100 μL) in the presence or absence of CH223191 (Selleckchem, TX, USA) or anti-ST2 (IL1RL1, receptor of IL-33, R&D Systems, Minneapolis, MN, USA) antibody. The medium of the lower chamber was replaced with a chemotactic medium containing a concentration of IL-33 (1 ng/mL, R&D Systems, Minneapolis, MN, USA). DEP (10 μg/mL) or IL-33 (10 pg/mL) was then added to the upper chamber and incubated overnight. The migration of HMC-1 cells was determined by counting the number of migrated cells in the lower chamber.

### RNA extraction, complementary cDNA preparation, and real-time reverse transcription-quantitative polymerase chain reaction (RT-qPCR)

After treatment, the total RNA of MCs was isolated using the RNeasy Mini Kit (Qiagen, Stanford, VA, USA) and then quantified using the NanoDrop™ 1000 spectrophotometer (Thermo Scientific, Waltham, MA, USA). cDNA was synthesized from 0.5 μg of total RNA through reverse transcription using the iScript™ cDNA Synthesis Kit (Bio-Rad Laboratories, Hercules, CA, USA), then quantified by RT-PCR using the Rotor-Gene™ 3000 system (Corbett Research, Sydney, Australia) using Tools SuperFast SYBR qPCR Reagent (Biotools, Taipei, Taiwan). Primer sequences are listed in Table 1. Data were analyzed further through Rotor-Gene 6 software (Corbett Research). The relative quantification of gene expression was normalized to the expression of glyceraldehyde-3-phosphate dehydrogenase.

**Table 1:** The primers designed for qPCR and ChIP

Primers	Sequences
qPCR	
IL-33 mRNA Forward	5′ GGTGTTACTGAGTTACTATG 3′
IL-33 mRNA Reverse	5′ GGAGCTCCACAGAGTCTTCCTTG 3′
GAPDH Forward	5′ AGGGCTGCTTTTAACTCTGGT 3′
GAPDH Reverse	5′ CCCCACTTGATTTTGGAGGGA 3′
ChIP	
IL-33 promoter Forward	5′ GCAAAGCTCTGCTAATGGAG 3′
IL-33 promoter Reverse	5′ CCAGAGCAATCATCTGCTAC 3′
Input GAPDH Forward	5′ AGGGCTGCTTTTAACTCTGGT 3′
Input GAPDH Reverse	5′ CCCCACTTGATTTTGGAGGGA 3′

### Enzyme-linked immunosorbent assay (ELISA)

Concentrations of IL-33 or other T2 cytokines (IL-4, cat. DY204; IL-5, cat. DY205; IL-13, cat. DY213) in conditioned media were measured by a specific human ELISA kit (R&D Systems, Minneapolis, MN, USA) according to manufacturer instructions.

### Western blotting

Expression of target proteins was measured by western blotting as described previously (Weng et al. 2018b). Briefly, HMC-1 cells were cultured in a dish with diameters of 6 cm or 10 cm, then treated with vehicle or DEP in the presence or absence of specific inhibitors. Whole-cell lysates (50 μg) were separated by a sodium dodecyl sulfate–polyacrylamide gel electrophoresis precast gel (Bio-Rad Laboratories, Hercules, CA, USA) and transferred onto polyvinylidene difluoride membranes (Bio-Rad Laboratories, Hercules, CA, USA). The immunoreactivity of proteins was detected by specific primary antibodies and followed by incubation with horseradish peroxidase-conjugated secondary antibodies and enhanced Chemiluminescence (ECL) substrate (Tools Super ECLHRP substrate, Biotools, TW) according to manufacturer instructions. Quantitative data were measured using a computing densitometer (Kodak, Tokyo, Japan).

### Co-immunoprecipitation

HMC-1 cells were grown in dishes of diameter 10 cm. Then, they were treated with vehicle or DEP (10 μg/mL) for 10 min. Nuclear protein fractions were separated according to the method described before (Yu et al. 2009). In brief, HMC-1 cells were washed with PBS and pelleted, then resuspended in hypotonic buffer containing 0.1% Nonidet P-40 (Sigma-Aldrich, MO, USA) for 15 min on ice. Nuclei were pelleted through centrifugation at 4100 × g for 1 min at room temperature, then washed with hypertonic buffer for 10 min on ice. After centrifugation at 6200 × g for 10 min at room temperature, nuclear pellets were lysed in 500 μL of pull-down buffer, then immunoprecipitated with a specific antibody against p65 (Santa Cruz, CA, USA) or the AhR (Santa Cruz, CA, USA) in the presence of protein A/G magnetic beads (Abcam, Cambridge, UK) at 4 °C overnight. Immunoprecipitated beads were washed thrice with a pull-down buffer. Samples were analyzed by western blotting with antibodies specific for the AhR or p65.

### Chromatin immunoprecipitation (ChIP)

ChIP assay was performed using the ChIP-IT Express Assay Kit (Active Motif, CA) according to the manufacturer’s instructions. In brief, after treated with 10 μg/ml DEP for 1 h, the HMC-1 cells (2 × 10^6^ cells) were cross-linked with formaldehyde at 37 °C for 10 min, followed by quenching the formaldehyde with 125 mM glycine. The cell lysates were then sonicated and centrifuged for 10 min at 15,000 × *g* at 4 °C to pellet the cell debris. The soluble cross-linked chromatins were immunoprecipitated overnight with anti-AhR, ARNT, or non-immune IgG antibodies. The DNA was subsequently purified and eluted with 50 μl elution buffer using the spinning filter and analyzed by quantitative PCR (qPCR). The sample DNA was normalized to input DNA. Primers were designed to detect the promoter region containing two NF-κB binding sites (620 bp), and sequences were listed in Table 1.

### Statistical analyses

The figures in the present study were conducted and statistically analyzed by GraphPad Prism 9. One-way analysis of variance (ANOVA), followed by Dunnett’s test, was used to evaluate the significance of the difference between mean values for the results of in vitro cell-line studies. A *p*-value of < 0.05 was considered statistically significant.

## Results

### DEP increased MC accumulation in the airways

**Fig. 1:**
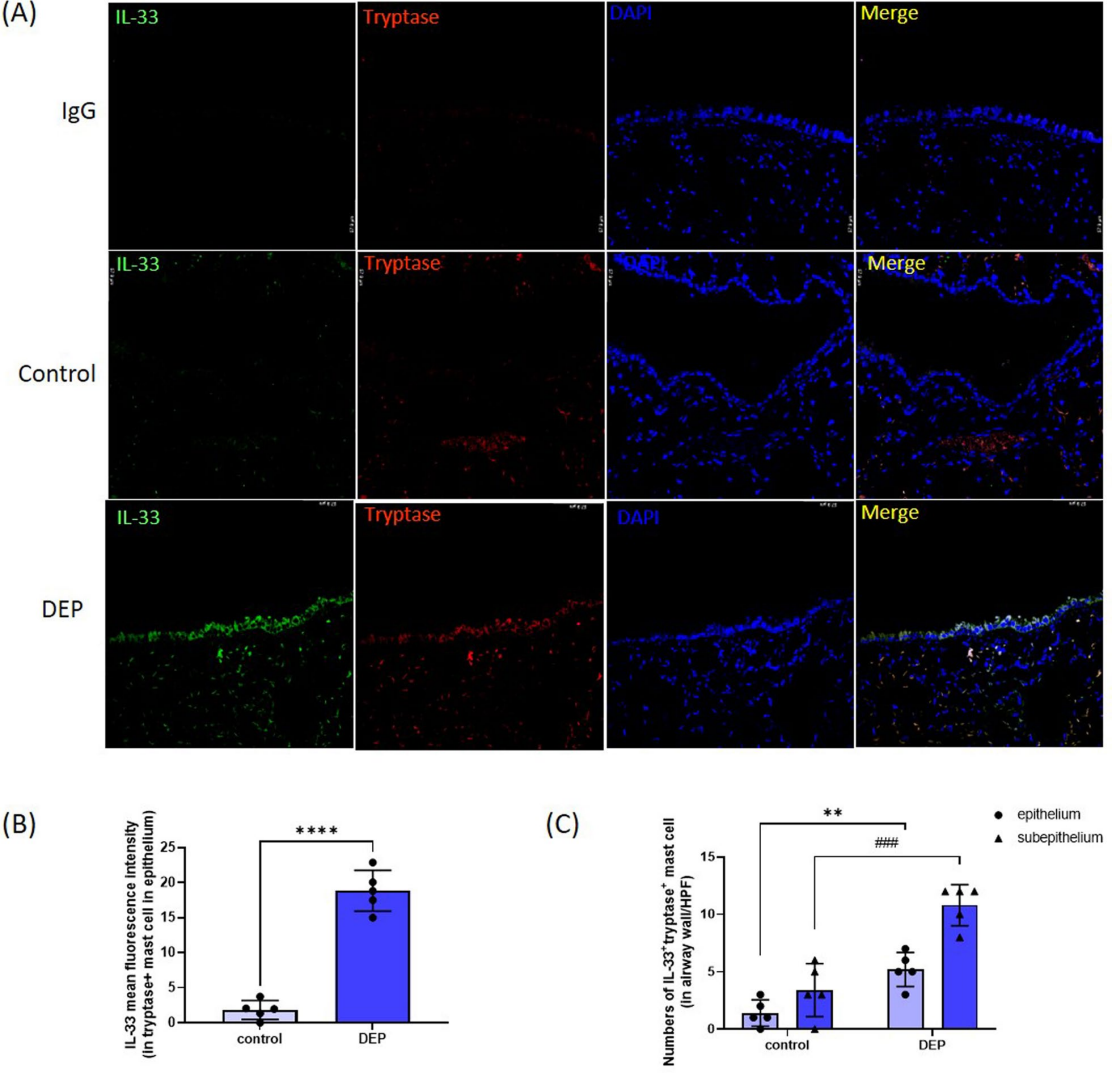
Increased number of mast cells in airway epithelium in DEP-challenge mice. Paraffin sections of airway tissues from animals with intra-tracheal instillation of DEP or vehicle (Control) were immunostained with specific antibodies for tryptase and IL-33 **a**. The statistical analysis showed DEP significantly increased the immunofluorescence intensity of IL-33 expression in tryptase^+^ MC **b** and the number of IL-33^+^tryptase^+^ MC in airway epithelium or sub-epithelial areas (**c**). Data are presented as means ± SEM. **p < 0.01; ****p < 0.001; ^###^*p* < 0.005; compared to the control

To determine whether DEP induces MC activation, mice were challenged with DEP through intratracheal instillation. As shown in Fig. 1, there were very few MCs infiltrated the airway epithelium or sub-epithelial areas in the control mice (Fig. 1a). Most MCs were found accumulated within blood vessels without expression of IL-33 (Fig. 1a). Intra-tracheal instillation of DEP increased the expression of IL-33 within tryptase^+^ MCs (Fig. 1b), and increased number of IL-33^+^tryptase^+^ MCs infiltrating the airway epithelium and sub-epithelial areas (Fig. 1c).

### DEP induced IL-33 release in human MC

**Fig. 2:**
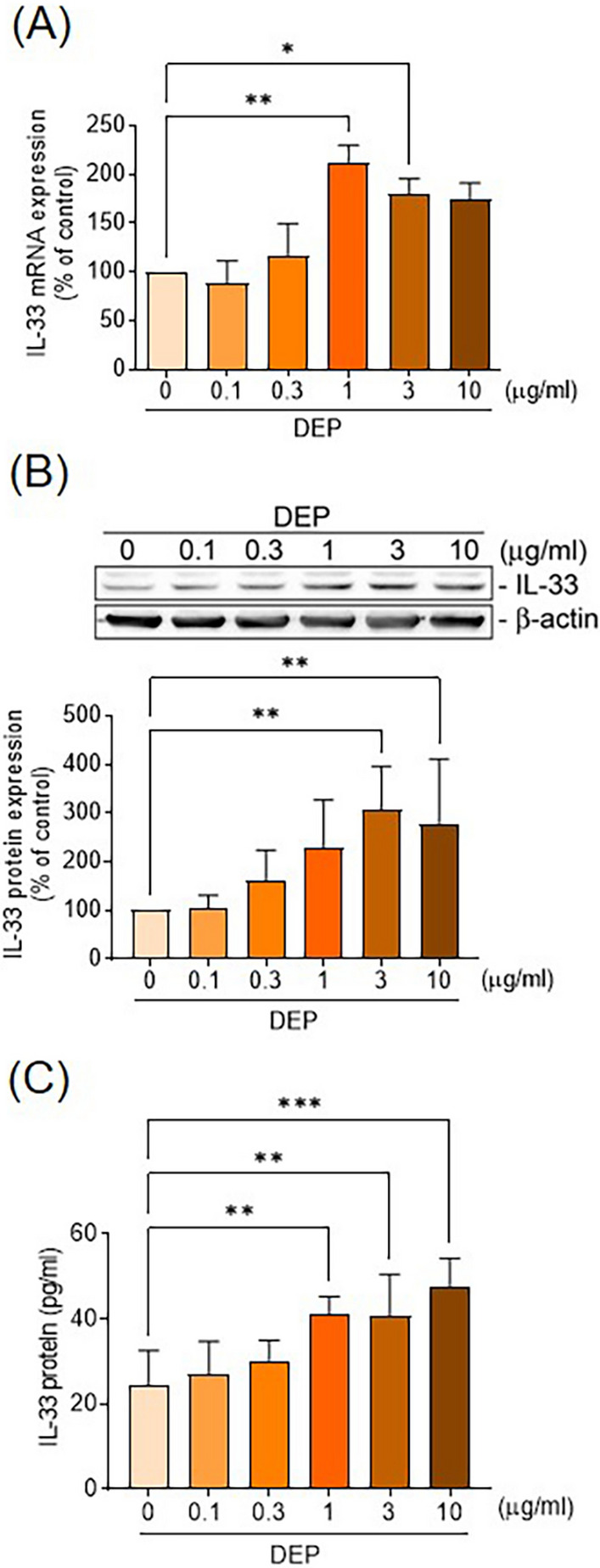
DEP induced IL-33 up-regulation in human mast cells. **a** HMC-1 cells were harvested after treatment of DEP (0.1–10 μg/ml) for 2 h, and IL-33 mRNA levels were assessed by quantitative PCR (N = 4). **b** HMC-1 cells were stimulated with DEP (0.1–10 μg/ml) for 6 h, then protein levels were analyzed by immunobloting for IL-33 using a specific antibody (N = 5). **c** HMC-1 cells were stimulated with DEP (0.1–10 μg/ml) for 24 h, and the levels of IL-33 in the conditioned medium were measured by ELISA assay (N = 5). Data are presented as means ± SEM from four (**a**) and five (**b**, **c**) independent experiments. *p < 0.05; **p < 0.01; ***p < 0.005 compared to the corresponding vehicle controls as the 100% reference

Our previous study demonstrated that DEP induces alarmin expression in airway epithelial cells from patients with severe asthma (Weng et al. 2018b). To investigate whether airborne pollutants induce IL-33 release in MC, HMC-1 cells were stimulated with DEP (0–10 μg/mL) for 2 or 6 h to measure IL-33 mRNA and protein expression. DEP stimulation induced HMC-1 cells to express the mRNA of IL-33 (N = 4) (Fig. 2a). Furthermore, DEP increased IL-33 protein expression in a concentration-dependent manner (N = 5) (Fig. 2b). To ascertain whether DEP induced IL-33 release, ELISA was performed on the culture media. Results revealed that IL-33 was produced in response to DEP in a concentration-dependent manner (N = 5) (Fig. 2c). These findings suggest that DEP induces the synthesis and release of IL-33 in HMC-1 cells.

### AhR mediated IL-33 expression in human MC

**Fig. 3:**
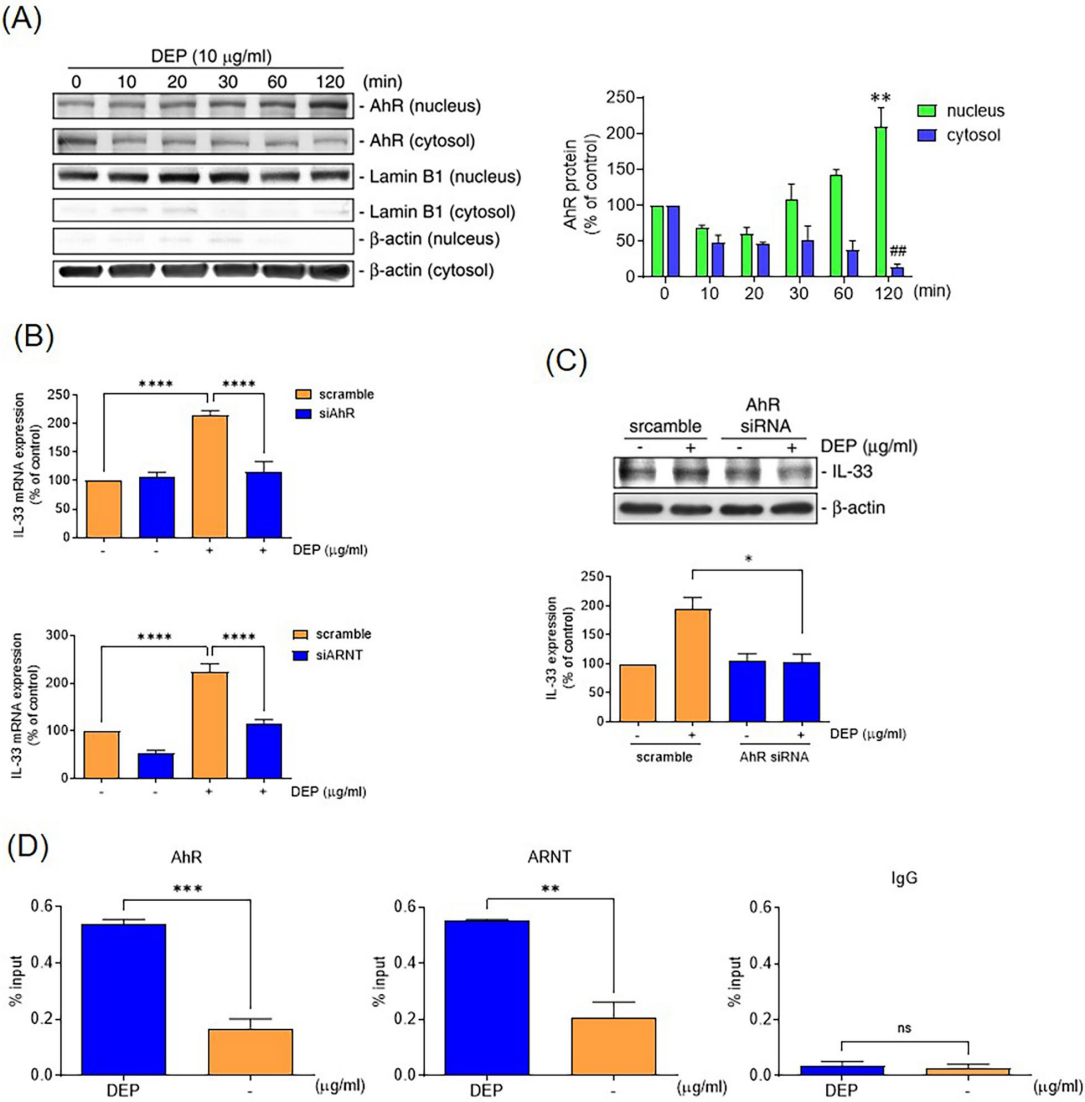
AhR mediated DEP-induced IL-33 mRNA expression in human mast cells. **a** HMC-1 cells were treated with DEP (10 μg/ml) for indicated time intervals, then harvested and separated into nuclear and cytosolic fractions. AhR protein levels were detected by immunoblot analysis. Lamin B1 and β-actin were used as an internal control for nuclear and cytosolic fractions, respectively (N = 3). Data are presented as means ± SEM from three independent experiments. **p < 0.01 and ##*p* < 0.01 compared to the corresponding baseline as the 100% reference. **b** HMC-1 cells were transfected with si*AhR* or si*ARNT* for 6 h, then stimulated with DEP (10 μg/ml) for 2 h. The mRNA levels of IL-33 were assessed by quantitative PCR as described above (N = 3). **c** Protein levels of IL-33 were detected by immunoblot analysis as described above (N = 3). **d** ChIP assay demonstrates DEP (10 μg/ml) increases the binding of AhR or ARNT to the IL-33 promoter sites (N = 3). Nonimmune IgG (IgG) was used as a negative control. Data are presented as means ± SEM of three experiments. * *p* < 0.05; **p < 0.01; ****p < 0.001 compared to the corresponding controls as the 100% reference

To investigate whether AhR is involved in DEP-induced IL-33 expression in HMC-1 cells, the cells were stimulated with DEP (10 μg/mL) for variuos time intervals, and nuclear and cytosolic fractions were isolated. AhR protein expression was observed in the cytosolic fraction 10 min after DEP stimulation, and became most prominent in the nucleus at 30 min, indicating nuclear translocation of AhR in response to DEP stimulation (N = 4) (Fig. 3a). Furthermore, HMC-1 cells were transfected with siRNA targeting AhR (si*AhR*) or ARNT (si*ARNT*), which almost completely inhibited DEP-induced mRNA expression of IL-33 (N = 4) (Fig. 3b) and DEP-induced expression of IL-33 protein (N = 4) (Fig. 3c). To determine whether AhR interacts with the endogenous IL-33 promoter region in response to DEP, a chromatin immunoprecipitation (ChIP) assay was performed. The results of the ChIP assay demonstrated that DEP increased the binding of the AhR and ARNT on the promoters of IL-33 (N = 3) (Fig. 3d). These results suggest that DEP induces AhR activation, which directly regulates the expression of IL-33 genes in HMC-1 cells.

### Role of nuclear factor-kappa B (NF-κB) activation in DEP-induced IL-33 expression of human MC

**Fig. 4:**
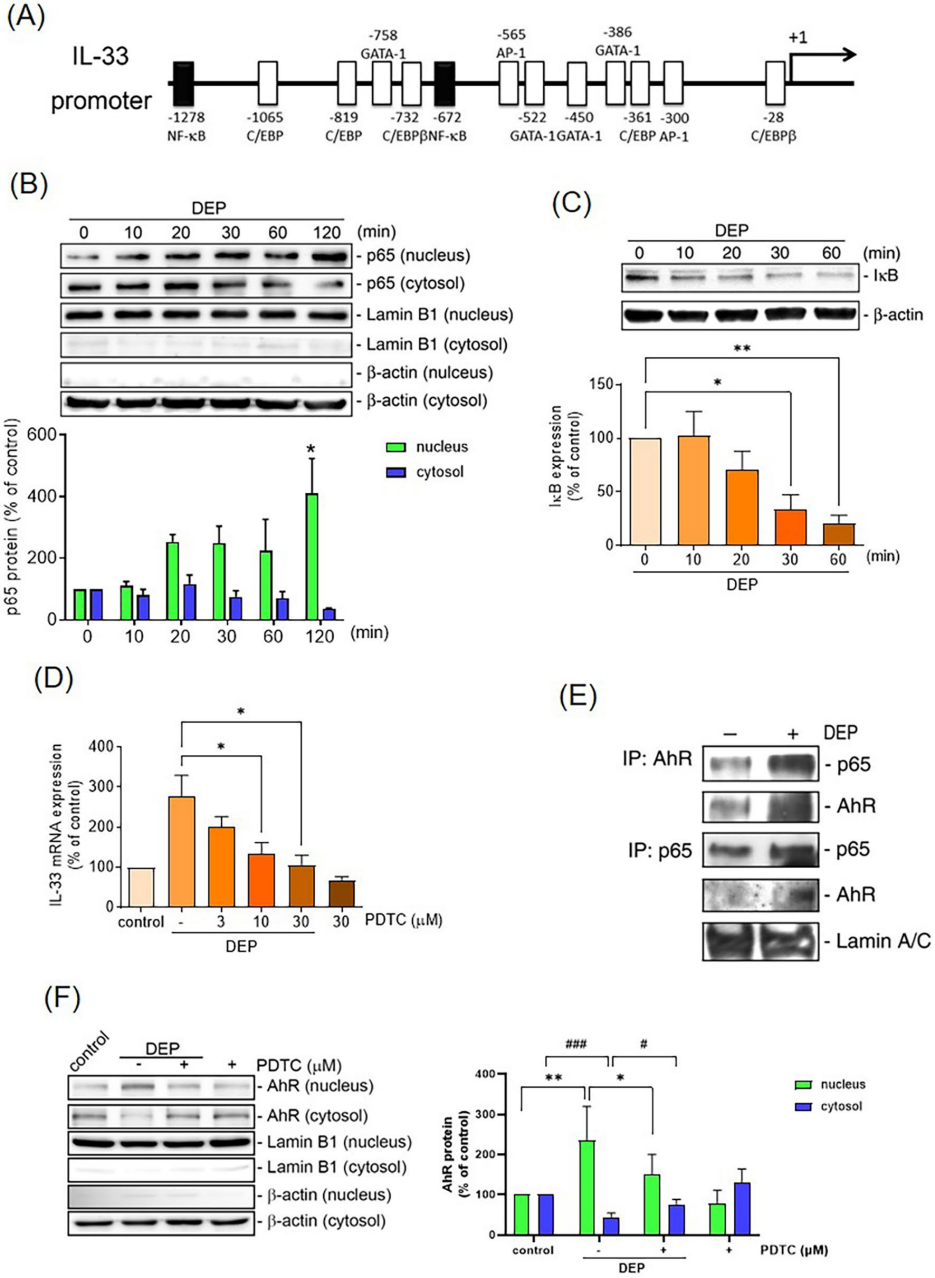
NF-kB activation facilitated AhR nuclear translocation in DEP-induced IL-33 expression of HMC-1 cells. **a** Schematic diagram of the human IL-33 promoter, with data collected from NCBI. **b** HMC-1 cells were stimulated with DEP (10 μg/ml) for the indicated time intervals, and the nuclear and cytosolic fractions were separated as described above. p65 protein levels were detected by immunoblot analysis. Lamin B1 and β-actin were used as internal controls for nuclear and cytosolic fractions, respectively (N = 4). **c** HMC-1 cells were stimulated with DEP (10 μg/ml) for the indicated time intervals, and cells were lysed and immunoblotted with antibodies specific for IκB or β-actin (N = 4). **d** HMC-1 cells were pretreated for 30 min with either equivalent vehicle control (DMSO) or PDTC (3–30 mM) and then stimulated with DEP (10 μg/ml) for 2 h. IL-33 mRNA levels were determined as described above (N = 4). Data are presented as means ± SEM from four independent experiments. *p < 0.05; **p < 0.01 as compared to DEP stimulation alone group. **e** HMC-1 cells were stimulated with DEP (10 μg/ml) for 30 min. The nuclear fraction was separated and immunoprecipitated with antibodies specific for AhR or p65 (N = 3). The immunoprecipitated complexes were immunoblotted for p65 or AhR. Lamin A/C served as the input control. Traces represent results from three independent experiments. **f** HMC-1 cells were stimulated with DEP (10 μg/ml) for 120 min in the presence or absence of PDTC (30 mM) (N = 5), and nuclear and cytosolic fractions were separated as described above. AhR protein levels were detected by immunoblot analysis. Lamin B1 and β-actin were used as internal controls for nuclear and cytosolic fractions, respectively (N = 5). Data are presented as means ± SEM from five independent experiments. *p < 0.05; **p < 0.01; #*p* < 0.05; ###*p* < 0.001 as compared to corresponding control group

AhR can interact with other transcription factors, such as NF-κB, to enhance gene expression (Kim et al. 2000). To determine whether NF-κB is involved in DEP-induced IL-33 expression in HMC-1 cells, cytosol/nucleus fraction isolation and co-immunoprecipitation assay were performed. Treatment with DEP (10 μg/mL) increased p65 translocation from the cytosol to the nucleus in a time-dependent manner (N = 4) (Fig. 4b), and induced IκBα protein degradation (N = 4) (Fig. 4c) in HMC-1 cells. Pretreatment of HMC-1 cells with a NF-κB inhibitor, PDTC (Ammonium pyrrolidinedithiocarbamate), significantly inhibited DEP-induced mRNA expression of IL-33 (N = 4) (Fig. 4d). Co-immunoprecipitation assay demonstrated that DEP induced the interaction between AhR and p65 (N = 3) (Fig. 4e). Furthermore, inhibition of NF-κB by PDTC resulted in a decrease in DEP-induced AhR nuclear translocation (N = 4) (Fig. 4f). These results indicate that DEP induces the activation of NF-κB, which interacts with AhR to facilitate nuclear translocation, leading to DEP-induced IL-33 expression in HMC-1 cells.

### IL-33 release from DEP-treated HMC-1 cells mediated the release of type-2 cytokines and migration of HMC-1 cells

**Fig. 5:**
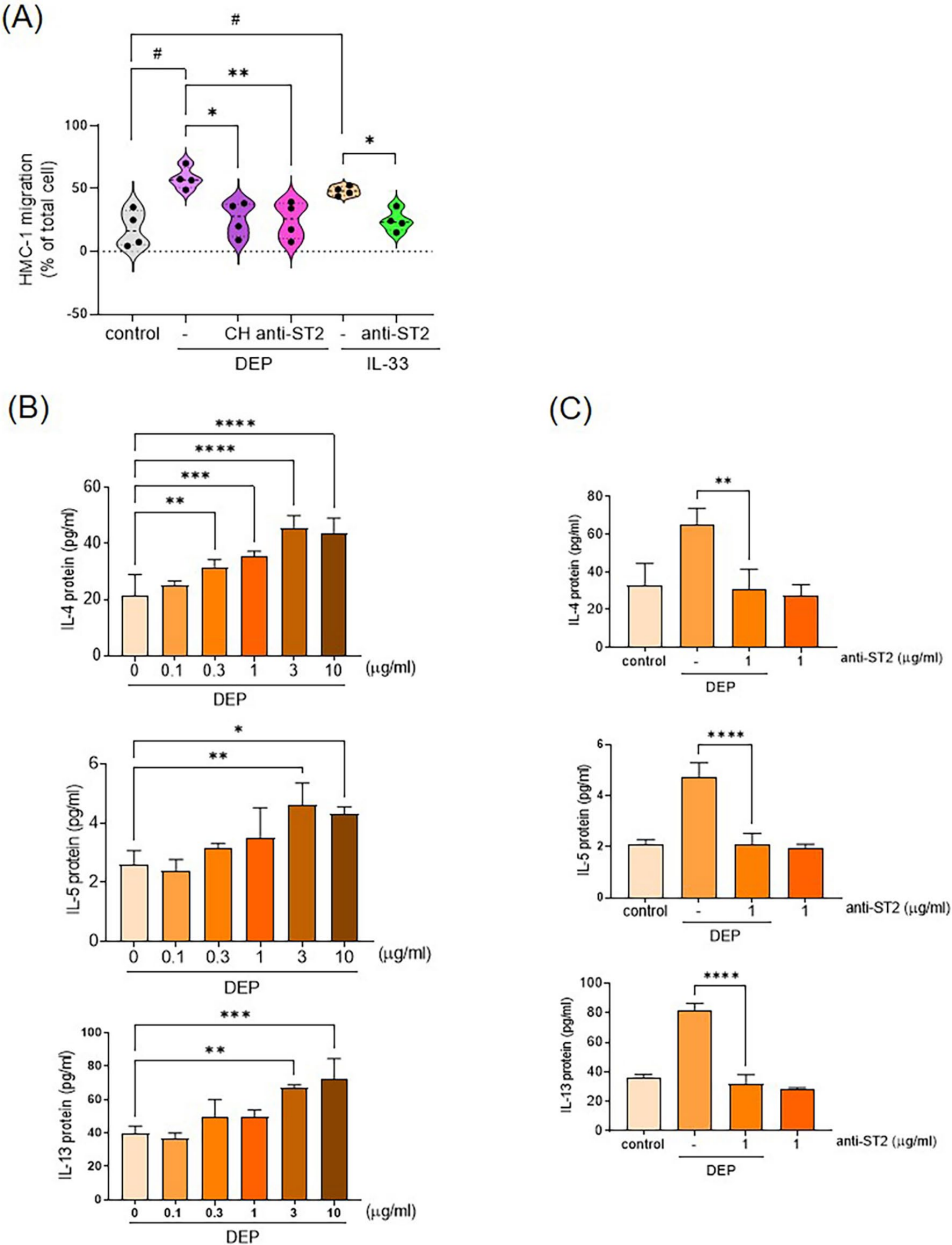
IL-33/ST2 axis involved in DEP-induced mast cell migration and type 2 cytokine release. **a** HMC-1 cells were cultured in the upper chamber of a Transwell system and incubated with CH223191 (CH, 10 μM) or an anti-ST2 blocking antibody (1 μg/ml), followed by stimulation with DEP (10 μg/ml) or IL-33 (10 pg/ml) for 24 h (N = 4). The migrated cells in the lower chamber were harvested and counted. Data are presented as means ± SEM from four independent experiments. *p < 0.05, **p < 0.01 compared to the corresponding vehicle controls; #*p* < 0.05 compared to the control group. **b** HMC-1 cells were harvested after stimulation with DEP (0.1–10 μg/ml) for 24 h, and the levels of IL-4, IL-5, and IL-13 were determined by ELISA (N = 4). **c** HMC-1 cells were stimulated with DEP (10 μg/ml) for 24 h in the presence or absence of an anti-ST2 blocking antibody (1 μg/ml). The levels of IL-4, IL-5, and IL-13 were subsequently determined by ELISA (N = 3). Data are presented as means ± SEM from four (**b**) and three (**c**) independent experiments. *p < 0.05; **p < 0.01; ***p < 0.005; ****p < 0.001 compared to DEP treatment alone

To investigate whether DEP induces MC migration, an in vitro migration assay was performed. Treatment with DEP (10 μg/mL) enhanced the transmembrane migratory ability of HMC-1 cells (N = 4) (Fig. 5a). This effect was inhibited by treatment with the AhR inhibitor CH223191 or anti-ST2 antibody (Fig. 5a). Therefore, the IL-33 expression induced by DEP in MCs is implicated in cell migration via the IL-33/ST2 axis. DEP also induced HMC-1 cells to release the type-2 cytokines, including IL-4, IL-5, and IL-13, in a concentration-dependent manner (N = 4) (Fig. 5b). Treatment with anti-ST2 neutralizing antibodies abolished DEP-induced type-2 cytokines release (N = 3) (Fig. 5c), suggesting that the IL-33/ST2 axis mediates DEP-induced type-2 cytokine release in HMC-1 cells.

## Discussion

MCs play a crucial role in the pathogenesis of asthma by releasing a broad spectrum of proinflammatory cytokines and chemokines that drive the immune responses, including the T2 response (Galli et al. 2008). Studies have shown that MCs can migrate to the airway epithelium, resulting in epithelial instability (Altman et al. 2019). In the present animal study, exposure to DEP increased IL-33^+^ MC infiltration into the airway epithelium and sub-epithelial areas, suggesting that direct exposure to air pollutants induces MCs to produce IL-33 and promotes airway epithelium-shifted migration. This inference is supported by our in vitro HMC-1 studies, which demonstrated that DEP directly induces IL-33 mRNA and protein expression in MCs through AhR and NF-κB activation. The increased IL-33 production mediated DEP-enhanced HMC-1 migratory ability and T2 cytokines expression, both of which were inhibited by anti-ST2 antibody treatment. These findings suggest that DEP exposure induces MC activation via the IL-33/ST2 axis in an autocrine or paracrine manner, and IL-33 plays a chemotactic role in MC migration.

MCs are abundant in the submucosa and generally occupy a perivascular position surrounding blood vessels in healthy controls (Lowman et al. 1988). In asthma, these MCs can shift from the submucosal compartment to the epithelium, a process strongly associated with type 2 inflammation (Altman et al. 2019). In our study, the vast majority of MCs in the control group of mice were found to accumulate within submucosal vessels, with very few infiltrating into the airway epithelium. Exposure to DEP not only enhanced IL-33 expression in MCs but also predominantly in airway epithelial cells. This finding aligns with our previous report that DEP induces the release of alarmins in airway epithelial cells of severe allergic asthma (Weng et al. 2018b). Therefore, DEP exposure may increase the migration of MCs into submucosal areas via autologous IL-33 produced by MCs, while also driving their chemotactic migration to the airway epithelium in response to epithelium-derived IL-33.

IL-33 promotes the differentiation of Th2 cells (Oliphant et al. 2011; Tamachi et al. 2006) and activates MCs and type 2 innate lymphoid cells (ILC2) (Oliphant et al. 2011), which subsequently trigger Th2-derived canonical cytokines. In the present study, DEP induced HMC-1 cells to release type-2 cytokines (IL-4, IL-5, and IL-13) via the IL-33/ST2 axis, consistent with previous findings (Altman et al. 2019). The increased levels of IL-5 in airway epithelium may induce trafficking and activation of eosinophils toward the airway epithelium (Johansson 2017). The elevated levels of IL-4 and IL-13 in the airway epithelium may contribute to dysfunction of the airway epithelial barrier (Saatian et al. 2013). These cytokines increase cellular permeability, leading to the disruption of tight junctions in the airway epithelium (Saatian et al. 2013), and play a role in the pathogenesis of airway remodeling in asthma (Vatrella et al. 2014). Furthermore, the epithelium-shifted IL-33^+ ^MCs, along with the production of type 2 cytokines, may create a feed-forward loop that amplifies the airway epithelium IL-33 expression (Christianson et al. 2015). Thus, we propose that exposure to airborne pollutants may lead to an excessive increase in IL-33 expression in airway epithelial cells and airway epithelium-shifted MCs. This, in turn, creates a vicious feedback loop involving type 2 cytokines production, airway epithelium disruption, and augmentation of airway inflammation in asthma.

As a recognized receptor for environmental pollutants, activation of the AhR plays an important role in regulating the expression of specific proinflammatory genes and mediating the differentiation of Th2 cells in asthma (Xia et al. 2015). Our previous study demonstrated that DEP induces alarmins release in airway epithelial cells of severe allergic asthma via AhR activation (Weng et al. 2018b). In the present study, we found that DEP also induces IL-33 expression in HMC-1 cells through AhR activation. These results indicate that exposure to airborne pollutants may induce excessive IL-33 expression, triggering an imbalance of the epithelium immune microenvironment and leading to severe T2 inflammation in asthmatic patients. Furthermore, DEP exposure also induced NF-κB activation in HMC-1 cells, and inhibition of NF-κB activation significantly reduced AhR nuclear translocation under DEP stimulation in HMC-1. The core functional region of the IL-33 promoter is located between − 1864 and + 77 bp upstream of the IL-33 gene (Govatati et al. 2019; Li et al. 2019). Using the gene sequence from the NCBI GEO database and TRANSFAC software, we identified two κB binding sites in the IL-33 promoter region (− 2000 to + 10 bp), but no DRE sites were detected (Fig. 5a). Our ChIP assay results demonstrated the recruitment of the AhR/ARNT complex to the IL-33 promoter region. Additionally, there are five potential NF-κB binding sites within the 3 kb proximal region of the IL-33 promoter. The NF-κB pathway has previously been shown to regulate IL-33 transcription in human endothelial cells (Duez et al. 2020). In this study, co-immunoprecipitation of nuclear fraction revealed that NF-κB interacts with AhR, suggesting that NF-κB activation may act as a chaperone for AhR nuclear translocation and that NF-κB is crucial in mediating DEP/AhR-mediated IL-33 expression in HMC-1 cells. Furthermore, the IL-33 promoter contains two dioxin response elements (DREs), which serve as AhR binding sites located in the distal region of the IL-33 promoter (Ishihara et al. 2019). These findings suggest that AhR/NF-κB activation may regulate IL-33 expression through promoter folding. Our previous study demonstrated that DEP induces NF-κB activation in airway epithelial cells via the ROS pathway (Weng et al. 2018a). However, the mechanism underlying DEP-induced NF-κB activation in HMC-1 cells needs further investigation.

The limitation of our study is that human primary mature MCs are notoriously difficult to obtain for research purposes. Consequently, many studies on human MCs use cells derived from differentiated MC progenitors in peripheral blood stimulated with IL-3. Although the differentiation protocol for mast cells is well-established, varying extents of cell differentiation may lead to differing outcomes in response to DEP stimulation. Although the HMC-1 cell line, derived from a patient with mast cell leukemia, is widely utilized in studies of human MC functions due to its expression of cytokines or cell surface antigen profile (Fu et al. 2021; Sundstrom et al. 2003), as well as for exploring the underlying regulatory molecular mechanism in allergic responses (Fang et al. 2022; Hu et al. 2024), the responses to DEP and the underlying regulatory mechanisms observed in HMC-1 cells need to be further validated using human primary MCs in the future studies.

## Conclusions

Airborne pollutants can enhance the expression of IL-33 in airway epithelium-shifted MCs via AhR/NF-κB activation. The concomitant increase in expression of IL-33 induces T2 cytokines production in MCs, potentially amplifying T2-mediated airway inflammation and contributing to the persistent exacerbation of severe asthma.

## Data Availability

The datasets used and/or analyzed during the current study are available from the corresponding author upon reasonable request.
